# The impact of medical insurance reimbursement on postoperative inflammation reaction in distinct cardiac surgery from a single center

**DOI:** 10.1186/s12913-022-07920-8

**Published:** 2022-04-13

**Authors:** Qin Jiang, Tao Yu, Keli Huang, Xiaobo Huang, Qingfeng Zhang, Shengshou Hu

**Affiliations:** 1 Department of Cardiac Surgery, Sichuan Provincial People’s Hospital, Affiliated Hospital of University of Electronic Science and Technology, No.32, West Second Section First Ring Road, Chengdu, 610072 China; 2 Department of Surgical Intensive Care Unit, Sichuan Provincial People’s Hospital, Affiliated Hospital of University of Electronic Science and Technology, Chengdu, China; 3 Department of Cardiovascular Ultrasound and Non-invasive Cardiology, Sichuan Provincial People’s Hospital, Affiliated Hospital of University of Electronic Science and Technology, Chengdu, China; 4 Department of Cardiac Surgery, Fuwai Hospital, National, Center for Cardiovascular Disease, Chinese Academy of Medical Sciences and Peking Union Medical College, Beijing, China

**Keywords:** Medical insurance reimbursement, Cardiac surgical procedures, Inflammation reaction indexes

## Abstract

**Background:**

Evidences shows that socioeconomic status is reversely associated with the risk of morbidity and mortality for people with cardiovascular disease via pro-inflammation mechanism, but the population profile is not deeply defined on. We aimed to investigate the impact of medical insurance coverage on postoperative systemic inflammatory reaction in two kinds of disease populations undergoing distinct cardiac procedures.

**Methods:**

A total of 515 patients receiving open mitral valve procedure with high-total expense from May 2013 through May 2021 in Sichuan Provincial People’s Hospital were retrospectively collected and stratified according to medical insurance reimbursement: low coverage with high out-pocket (< 30%), medium coverage (≤ 60%, but ≥ 30%), and high coverage (> 60%). Another 118 cases undergoing atrium septum defect (ASD) or patent foramen ovale (PFO) occlusion and taking on consistent low-total expense and low-coverage (< 30%) were also classified according to their insured conditions. The postoperative systemic inflammatory response indexes were high sensitivity C-reactive protein (hs-CRP) and the neutrophil–lymphocyte ratio (NLR).

**Results:**

Low insurance reimbursement population undergoing open mitral valve procedure had a higher level of hs-CRP and NLR but not troponin I protein or lactate within 48 h postoperatively, and higher thoracic drainage, longer ventilation use and stay in intensive care unit. No significant difference in inflammatory indexes existed among diverse medical insurance coverage in population undergoing ASD/PFO occlusion.

**Conclusions:**

Higher inflammatory reaction and weaker clinical recovery was associated with lower insurance coverage population undergoing open mitral valve procedure but not ASD/PFO interventional occlusion procedure.

**Supplementary Information:**

The online version contains supplementary material available at 10.1186/s12913-022-07920-8.

## Introduction

Socioeconomic status is broadly defined from the sociological and economic characteristics which impacts on an individual’s position in the society, and takes into account various factors such as income level, education attainment, occupation status, as well as environment factors. Socioeconomic status is regarded as important predictor for cardiovascular disease prognosis [1]. Low socioeconomic status is associated with elevated risk of complications and death for people with cardiovascular disease [2]. Furthermore, low socioeconomic status was associated with some health problems such as depression and cognitive impairment, which are characteristic of higher levels of inflammatory markers [3].

Although it was unlikely that it is an index that can accurately reflect socioeconomic status, previous study referred government’s medical insurance premium as a reference indicator [4]. In China, most of all cardiac procedure centers are accumulated in the municipality and provincial capitals, the severely suffered patients having cardiac procedure needs with rural household registration have to be transferred to the tertiary hospitals. However, the urban–rural segmentation of the medical insurance system makes the rural population benefit less reimbursement percentage of the total expenses than the urban residents [5]. A study conducted in western rural China revealed that high out-of-pocket spending and low healthcare reimbursement led to medical impoverishment, which was an important poverty source [6]. Under the background, some commercial insurance companies promise additional medical coverage if it is in the list of the covered disease items under the terms of the contract. There are some exceptions such as congenital heart diseases like secundum atrial septum defect (ASD) and patent foramen ovale (PFO), the total expense of occlusion procedure is relatively low and the compensations are also consistently limited among all the patients. That is why the occlusion device for closing the defect is not in the reimbursement catalogue if done in the adult period whatever medical insurance types. Therefore, we used the classification of medical insurance coverage percentage as the indicator of the socioeconomic status, and then, aimed to investigate the relationships between medical insurance reimbursement and postoperative inflammatory markers in the two populations with different diseases of distinct typical therapeutic mode.

## Methods

### Patients

This retrospective study was reviewed and approved by Human Research Ethics Committee of Sichuan Provincial People’s Hospital (No. 2021216) and conducted in accordance with the relevant guidelines and regulations of Declaration of Helsinki. The patients who were diagnosed as mitral valve disease or secundum ASD/PFO between May 2013 and May 2021 in Sichuan Provincial People’s Hospital were reviewed and informed consent was waived. All the included patients were above 18 years old and covered by Sichuan registration medical insurance. The etiologies on mitral valve of the selected cases included congenital, rheumatic and degenerative. However, if concomitant with more complex procedures such as other anomalies rectification, tumor removal, aortic valve or artery procedure, surgical atrial fibrillation radiofrequency ablation, coronary artery bypass grafting or emergency condition, the cases were screened out. The occlusion procedure was completed by transjugular transcatheter intervention using homemade device only under the surveillance of transesophageal echocardiography (TEE). The cases were also excluded out if the closure procedure failed and then converted to open repair. Other exclusion criteria included the conditions which have an impact on systemic inflammation, such as infection disease (infective endocarditis, active rheumatism), immunological disease (Behcet's disease, Takayasu's arteritis).

### Patient allocation

The subjects were classified into three groups based on the ratio of the medical insurance imbursement to the total hospitalized medical expenses for mitral valve procedure: group A-low coverage, less than 30%, typically covered by off-site new rural cooperative medical insurance with high out-of-pocket payments; group B-medium coverage, less than 60% but more than 30%, usually represented by urban population and government employee medical insurance, and group C-high coverage, more than 60%, additionally commercial health insurance based on group A or group B. It is important to note that the occlusion device was not covered by any insurance type, which usually accounted for about 3/4 of total hospital expenses. Therefore, these patients correspond to low coverage (less than 30%) but with low expenses.

### Anesthetic and operation scheme

All the included peoples received the homogeneous treatment and nursing care according to disease type. Anaesthesia was induced with midazolam, propofol, sufentanil, cisatracurium by endotracheal intubation and maintenance with propofol and sufentanil for mitral valve procedure. While, remifentanil replaced sufentanil for ASD occlusion procedure due to short half-time, if necessarily, with a low concentration of sevoflurane inhalation.

If necessarily, with a low concentration of sevoflurane inhalation, or remifentanil for ASD occlusion. All the patients receiving mitral valve procedure were conventionally transferred into intensive care unit (ICU) and then to general ward after surgical procedure. TEE-guided transcatheter ASD/PFO closure was completed via right jugular vein with dumbbell-shaped occlude device (Lifetech company, Beijing, China). These patients were extubated endotracheal tube after restoring to self-consciousness and transferred back to the surgical ward.

### Systemic inflammatory reaction indexes and other biomarkers

Blood samples were regularly drawn at the next dawn after the admission date, 12, 24, and 48 h after procedure for monitoring the postoperative conditions, from which the level of highly sensitive C-reactive protein (hs-CRP) was acquired, and the neutrophil–lymphocyte ratio (NLR) was calculated by dividing the absolute value of peripheral neutrophil by lymphocyte from complete blood count tests [7]. The troponin I (TnI) protein level was also collected at the same time-points. The artery blood gas was conventionally analyzed at the average interval of 8 h in the first 24 h postoperatively. The data on the lactate level was acquired from blood gas analysis record. The monitoring management for TnI was not requisite for ASD occlusion because severe coronary artery stenosis was excluded preoperatively out by coronary artery angiography if suspected. The detection results of the serum level of lactate after ASD occlusion was sporadic and almost of all were in normal limits.

### Clinical outcome measurements

Information on basic medical reimbursement status was collected from the final updated version in medical software system after the patient was discharged from the hospital. The additional insurance condition was enquired on the interviews at least one month after the hospitalization to ensure additional reimbursement results clear, including telephone conversation, internet consultation and conference participation [8].

The clinical data were collected from historical medical records, such as the durations of cardiopulmonary bypass (CPB), aortic cross-clamping (ACC) and the procedure administration, mechanical ventilation time, thoracic drainage volume, and the length of ICU and hospital stay [9].

### Statistical analysis

All statistical tests were two-tailed and associations were considered statistically significant if *p* ≤ 0.05 with SPSS software (version 17.0; SPSS Inc., Chicago, IL, USA). The distribution of data was assessed graphically (i.e., histograms), depending of normality, continuous variables were compared using the repeated measures ANOVA with post hoc comparisons to evaluate any significant effect of group or any significant interaction, differences between categorical variables were assessed using cross tabulation with chi-square analyses or the Fisher’s exact test. If there was no more than one piece of record missing in each item, a trend value was filled.

## Results

### Baseline data

**Fig. 1:**
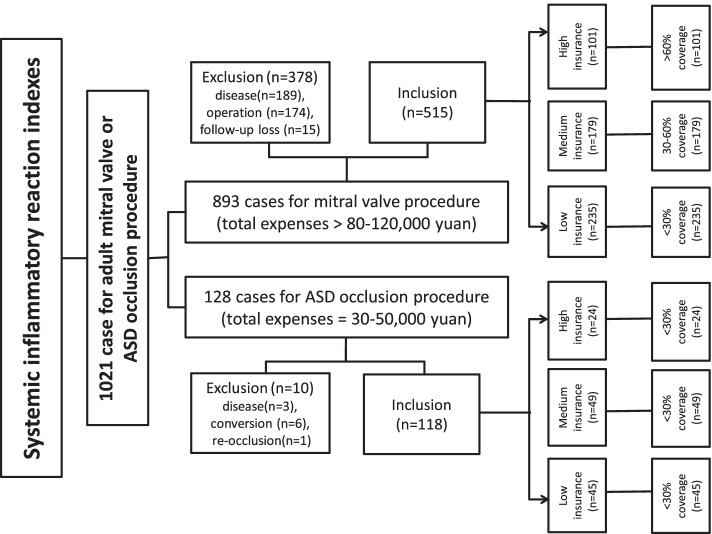
The study flowchart

Among 1021 adult patients of primary inclusion criteria with 893 cases for mitral valve procedure and 128 cases for ASD occlusion, 378 and 10 patients were excluded because of ineligible standards, additional operations or confounding diseases, respectively. As a final, a total of 235, 179 and 101 patients with mitral valve disease meeting the established criteria were recruited into group A, group B and group C, respectively. A total of 45, 49 and 24 patients with secundum ASD meeting our established standards were recruited into group A, group B and group C, respectively (Fig. 1). There were no significant differences in the baseline characteristics in the two populations, respectively (Tables 1  and 2).

**Table 1:** Baseline characteristics for mitral valve diseases

Characteristics	Group A (*n* = 235)	Group B (*n* = 179)	Group C (*n* = 101)	F/X^2^ value	^a^*P* value
Age, y	54.4 ± 11.2	54.3 ± 10.1	52.7 ± 10.3	0.952	0.387
Male (n, %)	65(46.4%)	65(46.1%)	38(37.6%)	2.254	0.324
Body Mass Index, kg/m^2^	21.9 ± 1.9	22.0 ± 2.0	22.3 ± 2.4	1.248	0.288
Current smoking (n)	18	11	13	4.042	0.133
drinking status (n)	7	9	5	1.336	0.513
inactivity status (n)	89	71	38	0.865	0.649
≥ High school Education level(n)	66	65	37	4.050	0.132
LVEF, %	56.7 ± 8.5	56.7 ± 7.7	57.2 ± 6.4	0.180	0.835
LVEDD, mm	52.4 ± 6.9	52.1 ± 6.7	51.6 ± 7.1	0.525	0.592
LAD, mm	52.4 ± 6.9	52.7 ± 6.3	53.8 ± 7.6	1.311	0.270
NYHA, I II III	33 107 95	18 86 75	10 54 37	3.143	0.534
Mitral valvuloplasty /replacement	89/146	72/107	36/65	0.600	0.741
Euroscore	2.0 ± 1.3	1.9 ± 1.3	2.0 ± 1.2	0.169	0.845

Group A: low medical coverage; Group B: medium medical coverage; Group C: high medical coverage

*BMI* Body Mass Index, *LVEF* left ventricular ejection fraction, *LVEDD* left ventricular end-diastolic dimension, *LAD* left atrium dimension, *NYHA* New York Heart Association

^a^*p* values obtained from the repeated measures ANOVA (continuous variables) and Pearson Chi-squared tests or Fischer’s exact (dichotomous variables)

**Table 2:** Baseline characteristics for atrial septum defect/ patent foramen ovale

**Characteristics**	**Group A** **(*n* = 45)**	**Group B** **(*n* = 49)**	**Group C** **(*n* = 24)**	**F/X**^**2**^** value**	^a^***P* value**
Age, y	42.1 ± 11.7	42.3 ± 11.8	42.4 ± 9.7	0.008	0.992
Male (n, %)	17(37.8%)	15(30.6%)	6(25.0%)	1.268	0.531
Body Mass Index, kg/m^2^, mean ± SD	23.3 ± 2.4	22.9 ± 2.5	22.8 ± 2.9	0.393	0.676
Current smoking	4	5	2	0.083	0.959
Current drinking	2	3	1	0.189	0.910
inactivity status	8	7	6	1.264	0.531
High school education	32	41	15	4.270	0.118
ASD/PFO	35/10	38/11	19/5	0.026	0.987
LVEF, %	60.1 ± 4.5	59.5 ± 5.4	59.8 ± 5.7	0.196	0.822
LVEDD, mm	41.6 ± 4.0	41.71 ± 4.1	42.0 ± 4.6	0.090	0.914
LAD, mm	40.1 ± 5.7	41.4 ± 4.7	40.2 ± 4.8	0.838	0.435

*ASD/PFO* atrial septum defect/ patent foramen ovale

### Postoperative hs-CRP, NLR, TnI and lactate level

**Fig. 2:**
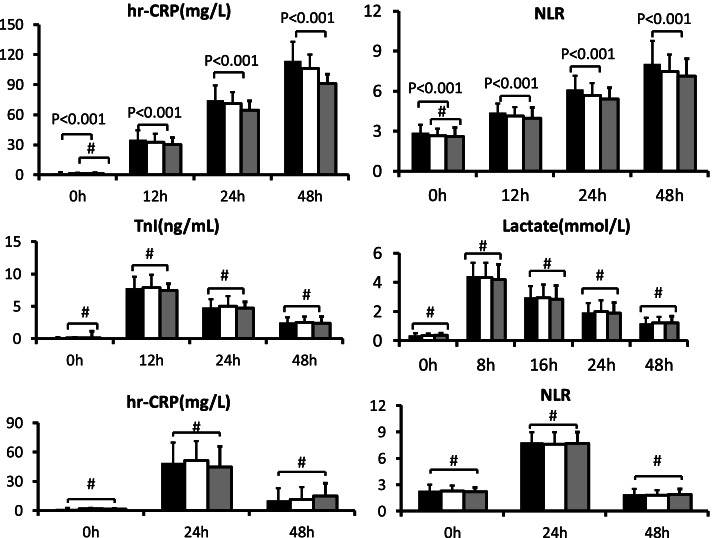
The inflammatory reaction and injury indexes in mitral valve and atrial septum defect occlusion procedure. Notes: The upper column indicated hr-CRP and NLR for mitral valve procedure. The middle column indicated TnI and lactate for mitral valve procedure. The lower column indicated hr-CRP and NLR for atrial septum occlusion procedure. The black bar indicated Group A: low medical coverage; the white bar indicated Group B: medium medical coverage; the green bar indicated Group C: high medical coverage

Serum levels of inflammatory factor hs-CRP and NLR value significantly increased after procedure. In the population of mitral valve procedure, the levels of hs-CRP and NLR at the postoperative 12 h, 24 h, 48 h were significantly higher in group A than those in group B and group C, respectively (Fig. 2, upper column). Marked elevation of TnI concentration above upper limit of norm was also observed in all groups post-operatively, but no significant difference of TnI was observed after procedure within 24 h among the three groups. The postoperative lactate level was also no remarked difference among the three groups in the four consecutive time-points, respectively (Fig. 2, middle column). However, in the population of ASD occlusion, the levels of hs-CRP and NLR at the postoperative 12 h, 24 h, 48 h were not significantly different among the three groups (Fig. 2, bottom column).

### Operative Outcomes and Survival

The postoperative outcomes and medical expenses for mitral valve procedure population were described in Table 3. The durations of CPB, ACC, and procedure administration were not significantly different among the three groups. There were more thoracic drainage volume and longer mechanical ventilation use in group A than those in group B and group C. The length of intensive care unit stay was longer in group A than those in group B and group C. No significant difference in hospital stay was also observed among the three groups. There was no in-hospital mortality during the 30-day follow-up period in all the groups. Only two patients in group B were re-hospitalized because of cerebral stroke after mitral valve procedure. There were no differences in procedure time and medical expenses among the population undergoing atrial septum defect occlusion procedure (Table 4).

**Table 3:** Intraoperative and postoperative parameters for mitral valve procedure

	**Group A** **(*n* = 235)**	**Group B** **(*n* = 179)**	**Group C** **(*n* = 101)**	**F value**	***P* value**
Total procedure time (mins)	196.8 ± 20.6	197.3 ± 20.9	199.6 ± 22.0	0.628	0.534
Bypass time(mins)	80.4 ± 15.5	77.9 ± 15.0	79.1 ± 16.2	1.344	0.262
Aortic cross clamp time (mins)	47.6 ± 12.3	46.5 ± 11.4	47.6 ± 13.1	0.480	0.619
Ventilation time (h)	13.8 ± 6.9	10.6 ± 5.4	9.7 ± 4.6	21.417	0.000
Chest drainage (ml)	700 ± 156	634 ± 153	632 ± 194	10.629	0.000
ICU stay (h)	23 ± 9	21 ± 9	20 ± 7	6.683	0.001
Hospital stay (d)	8 ± 1	8 ± 1	8 ± 1	1.080	0.340
Total medical expenses (thousand, yuan)	89.59 ± 7.26	89.43 ± 7.41	89.09 ± 7.79	0.162	0.850
Medical insurance reimbursement percentage	24.3 ± 2.2%	51.5 ± 3.0%	81.2 ± 6.6%	8629.341	0.000

Group A: low medical coverage; Group B: medium medical coverage; Group C: high medical coverage

*ICU* intensive care unit

**Table 4:** Intraoperative and postoperative parameters for atrial septum defect occlusion procedure

	**Group A** **(*n* = 45)**	**Group B** **(*n* = 49)**	**Group C** **(*n* = 24)**	**F value**	***P* value**
Total procedure time (mins)	27.4 ± 10.1	28.4 ± 11.2	27.8 ± 10.8	0.118	0.889
Hospital stay(d)	2.6 ± 1.1	2.6 ± 0.6	2.7 ± 1.3	0.167	0.846
Total medical expenses (thousand, yuan)	42.1 ± 2.4	41.7 ± 2.6	41.4 ± 2.9	0.703	0.497
Medical insurance reimbursement percentage	23.4 ± 3.0%	22.4 ± 2.6%	22.3 ± 3.2%	1.634	0.200

Group A: low medical coverage; Group B: medium medical coverage; Group C: high medical coverage

*ICU* intensive care unit

## Discussion

Our findings in the present study demonstrated that low versus high medical coverage percentage was associated with higher inflammation indexes and weaker intensive recovery among the population undergoing equivalent medical expenses for open mitral valve procedure. However, the phenomenon did not exist in the population undergoing transcatheter ASD occlusion procedure. To the best of our knowledge, the outcome added to the literature that negative correlation existed between medical insurance reimbursement and postoperative inflammation response undergoing cardiac procedure on cardiopulmonary bypass.

Psychosocial factors such as low socioeconomic status, acute or chronic stress and depression or anxiety are highly popular among the heart disease patients [10]. The patients with sepsis who live in the lowest median income populations had a higher risk of mortality and inflammation levels compared with the residents of the highest income [11]. Pro-inflammatory mechanism is regarded as a pivotal pathway translating socioeconomic inequalities into mental and physical health disparities [12]. Previous study showed that lower socioeconomic status independently predicted major adverse cardiac events via stress-associated neurobiological mechanism that includes heightened amygdalar activation, proinflammatory cytokine production from bone marrow immune cells, and atherosclerotic inflammation [13]. In the general child population, low socioeconomic status was associated with increased exposure to stressors like poverty and disadvantage, in turn associated with later inflammation [14]. Open mitral valve procedure need high medical expense, those who hold low medical insurance coverage were inclined to economic stress, reflected by psychological features such as anxiety and depression and concomitant higher inflammation indexes. In contrast, ASD interventional occlusion is exempt cardiopulmonary bypass and major surgical insult procedure. These patients enjoyed higher safety and only afforded less than a half of the total expenses which mitral valve procedure costs. Importantly, we did not observe remarked differences in baseline confounders of inflammation reaction in the recruited population like physical activities and adverse behavioral factors such as smoking, drinking, and obesity [15].

Inflammatory pathways provide important new intervention and prevention targets for cardiovascular and psychical disorders. There was robust evidence that easy-to-obtain blood-based inflammation biomarker indexed by hr-CRP significantly associated with sudden cardiovascular death risk in apparently low risk populations [16]. Higher level of systemic inflammatory markers such as CRP in the childhood was associated with an increased risk of developing depression and psychosis in young adulthood [17]. NLR is a non-specific, readily available blood-based biomarker of inflammation with potential scientific utility. Elevated NLR was associated with higher incidence, morbidity, and mortality in several systemic diseases, psychiatric, and age-related inflammatory conditions, including cardiovascular diseases and malignancies [18]. It was indicated in this study that medical reimbursement associated with postoperative inflammation response but not cardiac injury and energy metabolism indexed by TnI and lactate, respectively.

Low medical coverage usually indicated high out-of-pocket expenses during the cardiac procedure. In a cohort study, the individuals experiencing income volatility and drops were independently associated with a nearly twofold risk of cardiovascular disease and all-cause mortality [19]. In 2016, Chinese government launched healthcare reform including the new rural cooperative medical scheme, which the farmers, local finances, central government collectively raise the funds. The objective of this scheme enables the insured to enjoy the reimbursement benefit by paying a flat-rate premium. However, there is still a discrimination between the rural residents and urban employee in the present system of tiered medical services [20]. On account of no insurance coverage for occluder device which account for 75% of total hospital expenses, all of the insurance holders only shared with little reimbursement benefits from the rest part which was under the coverage. However, almost all of the expenses on open mitral valve procedure were included in the medical coverage. At last, the low medical coverage population could afford twofold expense of those that the high medical coverage population paid for. It indicated that economically affluent groups in the urban were more likely to receive advantageous finance reimbursements policy from independent insurance system, and meantime the reimbursements were also higher [21]. One study also showed that the implementation of essential medicines policies on primary healthcare institutions has certain positive but limited effects [22], which indicated that severe illness and impoverishment still occur like the twins in rural area [23]. Meanwhile, there were remarked difference in postoperative inflammation reaction among different medical insurance type only for mitral valve population but not for ASD occlusion population. Granted that the total expenses were relatively consistent for each cardiac procedure, the inflammation reaction was remarked elevated in serious cardiac procedure and reversely associated with practical medical payments.

Although there was no remarked difference on total hospital stay and expenses, weaker early intensive prognosis such as extended ICU stay and ventilation use predicts more negative emotions. Physical discomfort such as delayed chest tube removal will also trigger emotional abnormality that could result in compromised medical compliance [24]. Previous study showed that early sufficient physical excises is associated with a lower risk of death after cardiac valve procedure [25]. Moreover, over half of those who received ICU treatment were previously reported that significant symptoms like anxiety, depression or post-traumatic stress disorder appeared after discharge of hospital. Depression following critical illness is associated with an increased mortality risk in the first two years following discharge from ICU [26].

In previous studies, we investigated the series of the clinical conditions associated with the reduction of systemic inflammation reaction during cardiac procedure, such as remote ischaemic preconditioning [8], total thoracoscopic approach [27], and even high-altitude hypoxia environment [28]. In this study, we concentrated on the effect of medical insurance coverage in the specific population on inflammation reaction and clinical prognosis in the early period after heart surgery. The administration experiences on medicaid expansion remedies resulted in lower cardiovascular mortality in the middle-aged adults in United States [29]. Therefore, the equality on the benefits and compensations across diverse insurance coverage ranks in the population undergoing cardiac procedure with enormous expenditure should be prioritized on the basis of policy feasibility and equity. Meanwhile, more public and private sector efforts should be incorporated as part of a comprehensive strategy to alleviate medical coverage disparities. For the vulnerable populations, special policies such as covering the deficient medical insurance coverage on catastrophic procedure expenditure should also be considered. Implementation of the payment systems basedon Diagnosis-Related Group (DRG), which was established on the principle that same diagnoses and treatments require equal resource utilization, is also promising alternative [30].

The finding on the association between medical insurance coverage and postoperative inflammation response was limited due to the properties of observational study. The causality cannot be directly inferred from such retrospective study. Future studies focused on the validity of causality and mechanisms underlying the association between medical insurance coverage and postoperative inflammation are warranted. The attempt on completing the psychical stress questionnaire and commercial inflammatory cytokine detection should be considered in the future for further causality on the relationship.

### Public health implications

In all, the medical insurance coverage percentage was inversely associated with postoperative inflammation reaction in patient undergoing open mitral valve procedure but not ASD intervention occlusion. Although the financing of primary healthcare institutions was implemented in the past decade [31], more emphasis needs to be placed on the reimbursement gaps on the serious illness between urban and rural areas in the future. Given the current medical coverage of a wide distribution, improving understanding and awareness how to minimize the effect of medical imbursement distinction on clinical prognosis has become increasingly important.

## Supplementary Information

**Additional file 1:** **Supplemental file 1.** Entitled ASD was the data of the patients accepted atrium septum defect (ASD) or patent foramen ovale (PFO) occlusion.

**Additional file 2:** **Supplemental file 2.** Entitled MV was the data of the patients received mitral valve procedure.

## Data Availability

The datasets used in this study are provided and are available in the additional files.

## References

[1] Schultz WM, Kelli HM, Lisko JC (2018). Socioeconomic Status and Cardiovascular Outcomes: Challenges and Interventions. Circulation.

[2] Clark AM, DesMeules M, Luo W (2009). Socioeconomic status and cardiovascular disease: risks and implications for care. Nat Rev Cardiol.

[3] Koster A, Bosma H, Penninx BW (2006). Association of inflammatory markers with socioeconomic status. J Gerontol A Biol Sci Med Sci.

[4] Gwon JG, Choi J, Han YJ (2020). Community-level socioeconomic inequality in the incidence of ischemic heart disease: a nationwide cohort study. BMC Cardiovasc Disord.

[5] Wang Z, Chen Y, Pan T (2019). The comparison of healthcare utilization inequity between URRBMI and NCMS in rural China. Int J Equity Health.

[6] Wang Y, Zhu Y, Shi H (2019). The Effect of the Full Coverage of Essential Medicines Policy on Utilization and Accessibility of Primary Healthcare Service for Rural Seniors: A Time Series Study in Qidong, China. Int J Environ Res Public Health.

[7] Jiang Q, Liu S, Jiang L (2019). Comparison of two radiofrequency ablation devices for atrial fibrillation concomitant with a rheumatic valve procedure. Chin Med J (Engl).

[8] Jiang Q, Xiang B, Wang H (2019). Remote ischaemic preconditioning ameliorates sinus rhythm restoration rate through Cox maze radiofrequency procedure associated with inflammation reaction reduction. Basic Res Cardiol.

[9] Jiang Q, Yu T, Huang K (2018). Feasibility, safety, and short-term outcome of totally thoracoscopic mitral valve procedure. J Cardiothorac Surg.

[10] Albus C, Waller C, Fritzsche K (2019). Significance of psychosocial factors in cardiology: update 2018: Position paper of the German Cardiac Society. Clin Res Cardiol.

[11] Rush B, Wiskar K, Celi LA (2018). Association of inflammatory markers with socioeconomic status. Association of Household Income Level and In-Hospital Mortality in Patients With Sepsis: A Nationwide Retrospective Cohort Analysis. J Intensive Care Med.

[12] Muscatell KA, Brosso SN, Humphreys KL (2020). Socioeconomic status and inflammation: a meta-analysis. Mol Psychiatry.

[13] Tawakol A, Osborne MT, Wang Y (2019). Stress-Associated Neurobiological Pathway Linking Socioeconomic Disparities to Cardiovascular Disease. J Am Coll Cardiol.

[14] Kokosi T, Flouri E, Midouhas E (2020). Do upsetting life events explain the relationship between low socioeconomic status and systemic inflammation in childhood? Results from a longitudinal study. Brain Behav Immun.

[15] Bittoni MA, Wexler R, Spees CK (2015). Lack of private health insurance is associated with higher mortality from cancer and other chronic diseases, poor diet quality, and inflammatory biomarkers in the United States. Prev Med.

[16] Everett BM, Moorthy MV, Tikkanen JT (2020). Markers of Myocardial Stress, Myocardial Injury, and Subclinical Inflammation and the Risk of Sudden Death. Circulation.

[17] Khandaker GM, Pearson RM, Zammit S (2014). Association of serum interleukin 6 and C-reactive protein in childhood with depression and psychosis in young adult life: a population-based longitudinal study. JAMA Psychiat.

[18] Soder HE, Berumen AM, Gomez KE (2020). Elevated Neutrophil to Lymphocyte Ratio in Older Adults with Cocaine Use Disorder as a Marker of Chronic Inflammation. Clin Psychopharmacol Neurosci.

[19] Elfassy T, Swift SL, Glymour MM (2019). Associations of Income Volatility With Incident Cardiovascular Disease and All-Cause Mortality in a US Cohort. Circulation.

[20] Li J, Yuan B (2019). Rural-urban disparity in risk exposure to involuntary social health insurance transition in China: An investigation of chronic disease patients' mental health problems. Int J Health Plann Manage.

[21] Lai S, Shen C, Xu Y (2018). The distribution of benefits under China's new rural cooperative medical system: evidence from western rural China. Int J Equity Health.

[22] Guo Z, Guan X, Shi L (2017). The impacts of implementation of National Essential Medicines Policies on primary healthcare institutions: a cross-sectional study in China. BMC Health Serv Res.

[23] Sun M, Shen JJ, Li C (2016). Effects of China's New Rural Cooperative Medical Scheme on reducing medical impoverishment in rural Yanbian: An alternative approach. BMC Health Serv Res.

[24] Levine GN (2019). The Mind-Heart-Body Connection. Circulation.

[25] Kim SH, Cha S, Kang S, et al. High prevalence of physical inactivity after heart valve surgery and its association with long-term mortality: A nationwide cohort study. Eur J Prev Cardiol. Epub ahead of print 13 Feb 2020. DOI: 2020;2047487320903877. 10.1177/2047487320903877 33611453

[26] Hatch R, Young D, Barber V (2018). Anxiety, Depression and Post Traumatic Stress Disorder after critical illness: a UK-wide prospective cohort study. Crit Care.

[27] Jiang Q, Wang Z, Guo J (2021). Retrospective Comparison of Endoscopic Versus Open Procedure for Mitral Valve Disease. J Invest Surg.

[28] Jiang Q, Li H, Huang X (2020). Postnatal exposure to hypobaric hypoxia and its impact on inflammation and injury indexes after a cardiac valve procedure. Interact Cardiovasc Thorac Surg.

[29] Khatana SAM, Bhatla A, Nathan AS (2019). Association of Medicaid Expansion With Cardiovascular Mortality. JAMA Cardiol.

[30] Zhao C, Wang C, Shen C (2018). Diagnosis-related group (DRG)-based case-mix funding system, a promising alternative for fee for service payment in China. Biosci Trends.

[31] Shen C, Zhou Z, Lai S (2021). Whether high government subsidies reduce the healthcare provision of township healthcare centers in rural China. BMC Health Serv Res.

